# Decreased expression and function of sodium-glucose co-transporter 2 from a novel C-terminal mutation: a case report

**DOI:** 10.1186/s12882-016-0244-4

**Published:** 2016-03-21

**Authors:** Lei Yu, Qiaozhi Xu, Ping Hou, Hong Zhang

**Affiliations:** Renal Division, Inner Mongolia People’s Hospital, Hohhot, 010017 P.R. China; Computer & Information Engineering College, Inner Mongolia Normal University, Hohhot, 010022 China; Renal Division, Peking University First Hospital, Peking University Institute of Nephrology, Key Laboratory of Renal Disease, Ministry of Health of China, Beijing, 100034 China

**Keywords:** Familial renal glucosuria, SGLT2, *SLC5A2*

## Abstract

**Background:**

Familial renal glucosuria (FRG) is characterized by persistent glucosuria in the presence of normal serum glucose concentrations, and the absence of other impairments of tubular function. Mutations in the sodium–glucose co-transporter 2 (SGLT2) gene (*SLC5A2*) are causative of FRG the long-term outcome of which is well know. In the search for potential new drug targets for SGLT2 inhibitors with which to treat the diabetes, expressional and functional studies of SGLT2 have been the focus of attention, but reports of these are rare. Furthermore, it is well known that the alleles in the C-terminal are very important for the expression and function in some genes. However, little is known about the effect of mutation in *SLC5A2* C- terminal.

**Case presentation:**

Here, we identified a FRG patient with urine glucose excretion 7.56 g/day and a novel *SLC5A2* missense mutation, c.1891G > A/p.(E631K), by DNA sequencing. Expression and function of the mutant SGLT2 (631 K) fused to green fluorescent protein (GFP) were verified by western blotting, confocal laser microscopy, and transport activity assays in cultured HEK293 cells. Although wild-type SGLT2–GFP and 631 K mutant–GFP fusion proteins were properly expressed in a punctate pattern in the cell membrane, and co-localized with the cell membrane marker DiIC18(3), the expression of the mutant fusion protein was obviously decreased (24 %). Moreover, the uptake activity of the mutant SGLT2 631 K–GFP fusion protein was significantly decreased compared with wild-type (3629 ± 1082 vs. 7926 ± 1153, *P* < 0.001).

**Conclusion:**

These results suggest that the SLC5A2 C-terminal is very important for protein expression. We speculate that the observed reduced expression of the mutant transporter led to a decrease in transport of the glucose analog 2-(N-(7-nitrobenz-2-oxa-1,3- diazol-4-yl)amino)-2-deoxyglucose. The current study provides a starting point for further investigations of the SGLT2 molecular mechanism in FRG families, and offers functional insights into the development of anti-diabetes drugs.

## Background

Sodium–glucose co-transporter 2 (SGLT2) belongs to the Na + −glucose cotransporter family, and is a critical molecule in the process of glucose re-absorption from the urine in the proximal convoluted tubule [1]. The SGLT2 gene, *SLC5A2*, encodes 672 amino acids, is 7.7 kb long with 14 exons, and has been mapped to chromosome 16p11.2 [2]. The SGLT2 protein is located in the early proximal convoluted tubule, segment S1, and has a Na + −to-glucose coupling ratio of 1:1 [3]. Mutations in *SLC5A2* have been recently confirmed as responsible for the vast majority of familial renal glucosuria (FRG) cases [4, 5].

In a previous study, FRG patients were shown to have a good prognosis, so the principle behind SGLT2 inhibitor therapy is to improve diabetic conditions without increasing body weight or the risk of hypoglycemia [6, 7]. SGLT2 inhibitors have gradually become a research hotspot, but safety problems still hinder drug development [8]. For this reason, FRG patients are ideal models to search for pathogenic sites, and expression and functional studies of *SLC5A2* mutations may also enable novel SGLT2 drug targets to be identified. However, studies about the expression or function of mutations in FRG are rare, and the mechanism of action of mutations in the SGLT2 C-terminus is still unclear. This study reports a novel *SLC5A2* mutation in a FRG proband, and investigates its effect on SGLT2 expression and function using an in vitro system.

## Case presentation

The patient was a 39-year-old woman who was referred to the renal division because of repeated glucosuria. She had no polyuria, polydipsia, or weight loss. Her blood pressure was 120/70 mmHg, and her body weight was 55 kg. Routine urinary analysis showed 2+ to 3+ glucose with no other abnormalities. A quantitative test for urine glucose was 7.56 g/24 h. Her medical history and clinical examination revealed no significant findings. Fasting plasma glucose (4.92 mmol/l), albumin (42.8 g/l), creatinine (97 μmol/l), sodium (139.80 mmol/l), chloride (138.5 mmol/l), potassium (3.92 mmol/l), calcium (2.10 mmol/l), phosphate (1.04 mmol/l), magnesium (1.08 mmol/l), bicarbonate (19.4 mmol/l), uric acid (79 μmol/l), and hemoglobin A1C (5.3 %) were all within normal ranges. One hundred healthy Chinese volunteers (200 chromosomes) were included as controls. Informed written consent was obtained from all participants prior to participation in the study.

**Fig. 1:**
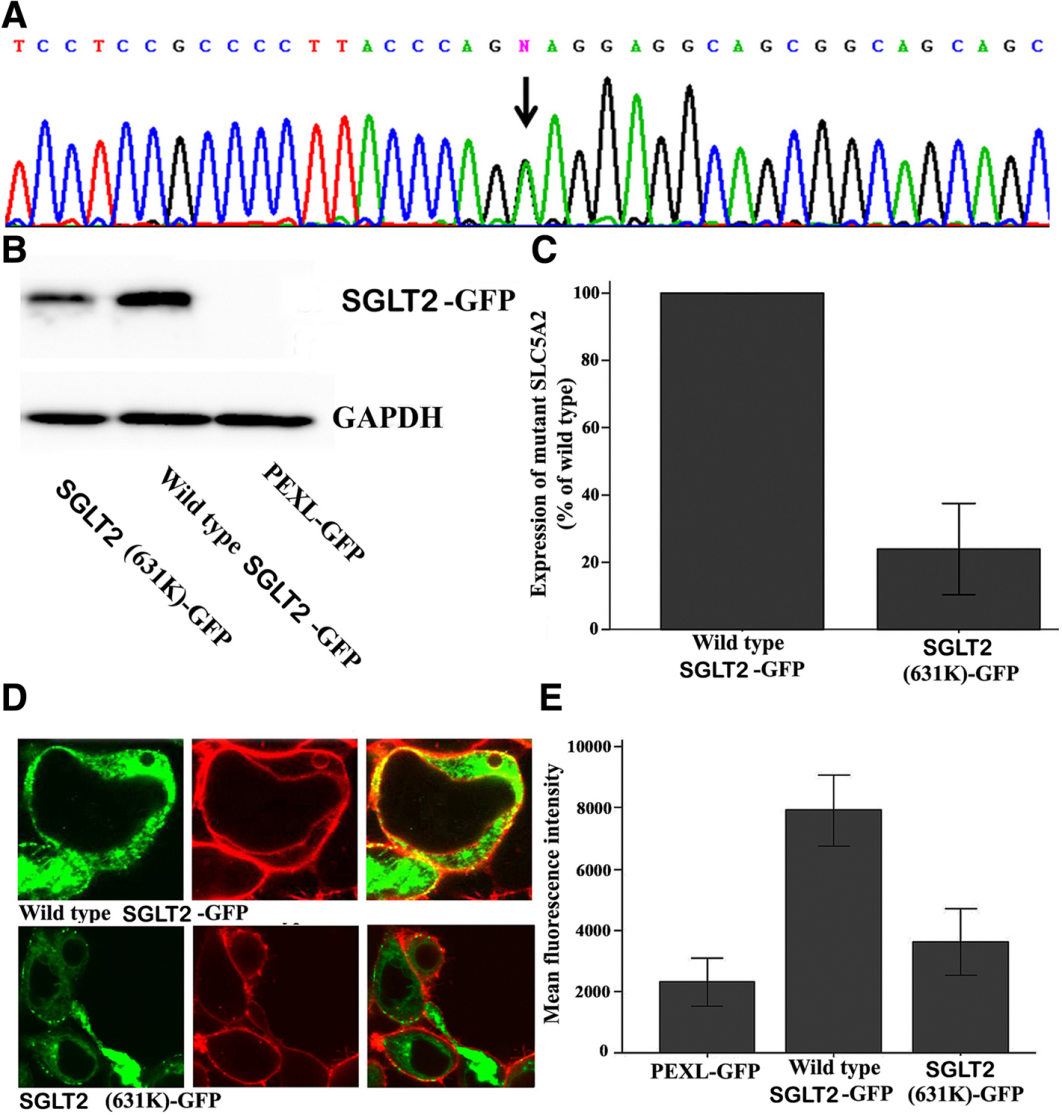
The expression and function of a novel SGLT2 C-terminal mutant. **a** The familial renal glucosuria patient carries a novel *SLC5A2* mutation (c.1891G > A/p.E631K). **b** Western blotting of wild-type and mutant SGLT2-GFP fusion proteins in 293 cells. **c** Expression levels of wild-type and mutant SGLT2-GFP. **d** Laser scanning confocal microscopy of wild-type and mutant SGLT2-GFP in 293 cells. **e** Transport activity of wild-type and mutant SGLT2-GFP in 293 cells

Genomic DNA was extracted by salting out from peripheral white blood cells. The entire coding region and adjacent intronic segments of *SLC5A2* were screened for mutations by the direct sequencing of PCR products. The genomic DNA reference sequences of *SLC5A2* (NG_012892.1, Gene ID: 6524, MIM: 182381, GEO Profiles ID: 62739973 and 65974292) and protein reference sequences of SGLT2 (NP_003032, UniProtKB - P31639) were acquired from the Entrez gene and protein database, respectively. To exclude the possibility that the identified mutations represented common polymorphisms, control chromosomes were tested by PCR-restriction-fragment length polymorphism. A novel missense *SLC5A2* mutation was found in the patient (c.1891G > A/p.E631K, Fig. 1a). The amino acid residue (631E) was found to be highly conserved among human SGLT subtypes and across SGLT2 homologs in multiple species. The mutation was not detected in any of the control 200 chromosomes, indicating that it does not represent a common polymorphism.

Human *SGLT2* cDNA from normal kidney, generated by reverse transcription (RT)-PCR, was cloned into the pGEM-T easy vector (Promega, Madison, WI). Wild-type and c.1891A mutagenized *SLC5A2* generated by site-directed mutagenesis were subcloned into the PEXL-GFP vector [9], and verified by sequencing. Human HEK293 cells (obtained from central laboratories of Peking Union Medical College Hospital, and originally from the American Type Culture Collection) were seeded into 24-well plates 24 h before transfection. Plasmid constructs (0.5 μg) were transfected into cultured cells at 70–80 % confluency using X-tremeGENE HP DNA transfection reagent according to the manufacturer’s instructions (Roche, Mannheim, Germany). After 24 h of incubation, expression of SGLT2 wild type–GFP and mutant–GFP fusion proteins was detected by western blotting, confocal laser microscopy, and transport assays as we have done in previous studies [10, 11].

Western blotting analysis (Fig. 1b) demonstrated that 631 K SGLT2 expression was significantly lower than that of wild-type SGLT2–GFP (0.24 ± 0.14 vs. 1, *P* = 0.002, *n* = 4, Fig. 1c). Confocal imaging revealed that both the wild-type and mutant fusion proteins were expressed in a punctate pattern in the cell membrane, which merged well with the cell membrane marker 1,10-dioctadecyl-3,3,3’,3’-tetramethyl-indocarbocyanine perchlorate (DiIC18(3), Fig. 1d).

The function of wild-type SGLT2–GFP and 631 K SGLT2–GFP fusion proteins in cultured HEK293 cells was confirmed by transport of the glucose analog 2-(N-(7-nitrobenz-2-oxa-1,3- diazol-4-yl)amino)-2-deoxyglucose (2-NBDG), evaluated by fluorescence intensity using flow cytometry. 2-NBDG uptake in HEK293 cells transfected with wild-type SGLT2–GFP was increased about 3.5-fold compared with those transfected with GFP-only control vector (7926 ± 1153 vs. 2314 ± 791, *P* < 0.001, *n* = 4, Fig. 1e). The uptake activity of the mutant SGLT2 631 K-GFP fusion protein was significantly decreased compared with wild-type SGLT2–GFP (3629 ± 1082 vs. 7926 ± 1153, *P* < 0.001, *n* = 4, Fig. 1e).

The Medical Ethics Committee of the Inner Mongolia People’s Hospital and Peking University approved the protocol. Data were compared with the *t*-test or analysis of variance followed by Fisher’s least significant difference methods for multiple comparisons. Data are shown as the mean ± SD, and significant differences were declared at *P* < 0.05.

Kidney proximal tubules reabsorb almost 180 g glucose daily, which is filtered through the glomeruli. SGLT2 accounts for most glucose reabsorption. It is responsible for the active transport of glucose across the brush border membrane, and is expressed almost exclusively in the kidney [1]. Recent studies have reported that *SLC5A2* mutations are causative of FRG [4, 5, 10–12]. The long-term outcome of FRG patients is excellent, so SGLT2 inhibitors have been the subject of particular attention for the treatment of diabetes [6, 7]. Although research into FRG may help with a breakthrough for diabetes treatment, expression and functional studies of *SLC5A2* mutations in FRG are rare, and the role of SGLT2 C-terminal mutations needs further clarification.

A previous study [4] suggested that overt glucosuria requires the individual to be homozygous or compound heterozygous for *SLC5A2* mutations. Consistent with this, our patient with a heterozygous mutation had “mild” glucosuria (urine glucose excretion, 7.56 g/day). However, our findings still imply that the mutation causes a clinically relevant SGLT2 dysfunction. Our in vitro study of 293 cells showed that the transport activity of the mutant SGLT2 631 K-GFP fusion proteins was significantly lower than that of wild-type. *SLC5A2* mutations may reduce or abolish transporter activity by impairing protein synthesis, processing, or insertion into the plasma membrane. Furthermore, transporter activity may be reduced or abolished by accelerating protein removal or degradation, altering functional regulation, or impairing intrinsic activity. The mutant SGLT2 631 K–GFP in our study had a similar punctate membrane expression pattern to wild-type, but a decreased expression intensity. Thus, the mutation (c.1891G > A/p.E631K) is likely to impair protein synthesis or accelerate protein removal or degradation. Our results also suggest that the SGLT2 C-terminal is very important for protein expression levels. We speculate that the reduced expression of the SGLT2 mutant is responsible for the decreased transport activity of mutant SGLT2.

Although it was not possible to perform a family study in the present case, identification of mutations that cause glucosuria will enable the establishment of a genotypic FRG diagnosis, providing important information for families and physicians. In general, renal biopsies are unnecessary for FRG patients, so the effect of the 631 K mutation on SGLT2 expression in the kidney is still unknown. We were restricted to an in vitro study of the expression and function of SGLT2 using GFP as a fluorescent label because we previously found that the SGLT2 antibody was unsuited to use in 293 cells, COS-7 cells, and *Xenopus laevis* oocytes.

## Conclusions

In summary, we identified a novel *SLC5A2* mutation in a Chinese FRG patient. Upon reconstruction in cultured cells, the SGLT2 C-terminal mutant protein showed reduced expression intensity and glucose transport capacity compared with wild-type. The mutant transport function may be reduced because of low mutant protein expression. Our study provides valuable information about the SGLT2 molecular mechanism which will be useful for anti-diabetes drug screening.

### Consent

Informed written consent was obtained from all participants prior to participation in the study. Written informed consent was obtained from the patient for publication of this Case report. A copy of the written consent is available for review by the Editor of this journal.

## Abbreviations

DiIC18(3): 1,10-dioctadecyl-3,3,3’,3’-tetramethyl-indocarbocyanine perchlorate
FRG: familial renal glucosuria
GFP: green fluorescent protein
2-NBDG: 2-(N-(7-nitrobenz-2-oxa-1,3- diazol-4-yl)amino)-2-deoxyglucose
PCR: polymerase chain reaction
SGLT2: sodium-glucose co-transporter 2
*SLC5A2*: sodium-glucose co-transporter 2 gene
